# MicroRNA expression associated with low-grade cervical intraepithelial neoplasia outcomes

**DOI:** 10.1007/s00432-023-05023-3

**Published:** 2023-07-08

**Authors:** Ashley N. Winters, Alex K. Berry, Tracy A. Dewenter, Nowrin U. Chowdhury, Kelly L. Wright, Jennifer E. Cameron

**Affiliations:** 1 Department of Microbiology, Immunology and Parasitology, Louisiana State University Health Sciences Center, 1901 Perdido St., New Orleans, LA 70112 USA; 2 Department of Pathology, Louisiana State University Health Sciences Center, 1901 Perdido St., New Orleans, LA 70112 USA; 3 The Stanley S. Scott Cancer Center, Louisiana State University Health Sciences Center, 1901 Perdido St., New Orleans, LA 70112 USA; 4 Present Address: Department of Microbiology and Immunology, Tulane University School of Medicine, 1430 Tulane Ave. 8638, New Orleans, LA 70112 USA; 5 Present Address: Department of Pathology, Microbiology and Immunology, Vanderbilt University, 1161 21St Ave S Medical Center North T-2219, Nashville, TN 37232 USA; 6 Present Address: Medical Science Liaison, Gynecologic Oncology, AstraZeneca. 1 Medimmune Way, Gaithersburg, MD 20878 USA

**Keywords:** Cervical intraepithelial neoplasia, Low-grade cervical dysplasia, Cervical dysplasia progression, MicroRNA, Wnt signaling

## Abstract

**Purpose:**

Only a fraction of low-grade cervical intraepithelial neoplasia (CIN) progresses to high-grade CIN; however, the biological processes that differentiate progressive CIN from CIN that resolves naturally are poorly understood. MicroRNAs (miRNAs) are important epigenetic regulators of gene expression and thus, miRNA expression profiling can reveal the dysregulated biology underlying disease processes. The purpose of this case–control study was to reveal miRNA expression patterns and predict the underlying biological pathways that are associated with clinical outcomes of low-grade CIN.

**Methods:**

Women with low-grade CIN diagnosis and definitive clinical outcomes (*n* = 51) were identified retrospectively using electronic clinical records. Comprehensive miRNA expression profiling was performed on the low-grade CIN diagnostic cervical biopsies retrieved from pathology archives. Differential miRNA expression was analyzed by comparing women with CIN that progressed to women with CIN that resolved naturally.

**Results:**

Differential expression of 29 miRNAs was observed in low-grade CIN that progressed to high-grade compared to low-grade CIN that resolved. Of these, 24 were significantly downregulated in progressive CIN, including miR-638, miR-3196, miR-4488, and miR-4508, while 5 miRNAs, including miR-1206a, were significantly upregulated. Computational gene ontology analysis based on the discovered miRNAs and their putative mRNA targets revealed biological processes associated with oncogenic phenotypes.

**Conclusion:**

Distinct miRNA expression profiles are associated with clinical outcomes of low-grade CIN. The functional effects of the differentially expressed miRNAs may be biological determinants of CIN progression or resolution.

**Supplementary Information:**

The online version contains supplementary material available at 10.1007/s00432-023-05023-3.

## Introduction

Cervical cancer screening and management guidelines published by the United States Preventive Task Force on Cervical Cancer Screening (2018), widely endorsed by professional societies, rely on a combination of cytology, human papillomavirus (HPV) testing, colposcopy and tissue biopsy to determine appropriate clinical management of women participating in screening (Force et al. 2018). This screening program has been monumental in reducing cervical cancer incidence in the U.S. through treatment intervention in women with high-grade cervical intraepithelial neoplasia (HGCIN). A byproduct of this highly effective cancer prevention program is that, in previously published estimates, between $1 and 2 billion USD in healthcare expenditures was attributed to the follow-up management of women with screen positive tests (Chesson et al. 2012), and nearly a quarter of a million U.S. women were diagnosed with low-grade cervical intraepithelial neoplasia (LGCIN) (Henk et al. 2010). Vaccine uptake and changes to screening guidelines (HPV co-testing and increasing screening intervals for some women) have undoubtedly impacted these estimates in more recent years; however, one thing remains clear: low-grade dysplasia resolves without treatment for most women and progresses to high-grade dysplasia in fewer than one in ten women (Baseman and Koutsky 2005). While treatment intervention is compulsory for HGCIN, the clinical management algorithm for LGCIN remains complex and relies heavily on management-by-observation approaches with repeat screening tests at short-term intervals. It has been reported that on average, women with a LGCIN diagnosis undergo between 6 and 8 clinical encounters to monitor their dysplasia (Henk et al. 2010; Insinga et al. 2004). This observational approach has undesirable consequences. Repeat patient encounters in women with dysplasia poses risks for adverse effects (pain, cramping, bleeding, vaginal discharge, infection, dysmenorrhea and scarring associated with coloposcopy/biopsy) and psychosocial trauma (Gray et al. 2006; Sharp et al. 2009; McCaffery et al. 2010). Further, this approach delays treatment for those women who will experience dysplasia progression, creating the potential to miss the opportunity to intervene and prevent cancer development. The latter is of particular concern, as a significant proportion of cervical cancer cases can be attributed to inadequate management of dysplasia despite adequate screening (Spence et al. 2007). This failure in the cancer prevention care continuum is exacerbated in populations experiencing barriers to healthcare access that interfere with adherence to follow-up care, thereby contributing to socioeconomic and racial/ethnic disparities in cervical cancer incidence (Musselwhite et al. 2016). Notably, observational management guidelines were developed to address concerns of over-treatment of dysplasia in the absence of strong predictive biomarkers of LGCIN progression and pharmaceutical interventions that promote resolution of LGCIN. A better understanding of the biology underlying progression of LGCIN is needed to address these limitations in the cervical cancer prevention program.

MicroRNAs (miRNAs) are ~ 21 nucleotide, non-coding RNAs that regulate protein expression through binding of cognate sequences in the regulatory regions of mRNA transcripts. Dysregulation of miRNA expression is observed in disease states, and disruption of the miRNA-associated epigenetic regulatory mechanisms is often associated with loss of tumor suppressor function or gain of oncogene expression in solid tumors (Chen et al. 2012). MicroRNA dysregulation has been observed in cervical cancer and CIN2 + compared to healthy cervical tissue (Cheung et al. 2012; Wilting et al. 2013). Further, miRNA dysregulation has been observed in pre-neoplastic conditions and has demonstrated the potential to reveal cellular pathways involved in disease progression (Oberg et al. 2011; Stachowiak et al. 2017; Craig et al. 2020). To explore the miRNA regulatory pathways associated with cervical dysplasia progression, we conducted a case–control study in which we performed comprehensive miRNA expression profiling in low-grade cervical dysplasia tissue specimens from women with observed clinical outcomes. By comparing the miRNA expression profiles obtained for women whose low-grade dysplasia resolved naturally to that of women whose low-grade dysplasia progressed to high-grade, we identified miRNA dysregulation associated with dysplasia progression and revealed cellular pathways potentially involved early in advancement of cervical pre-neoplasia.

## Methods

### Screening of candidates for the study

Women who attended the University Medical Center/Interim LSU Hospital (New Orleans, Louisiana, USA) for cervical cancer screening from 2006 to 2018 were considered for inclusion in this study. Women with a recorded diagnosis of low-grade cervical intraepithelial neoplasia (LGCIN/CIN1) were screened for eligibility as a case (women with LGCIN and subsequent high-grade cervical intraepithelial neoplasia (HGCIN/CIN3)) or control (women with LGCIN and evidence of resolution without medical intervention). Two methods were employed to capture suitable candidates. The first method, which pre-dated the implementation of the hospital electronic medical record system, relied upon query of an electronic database of archived pathology specimens. A list of potential study candidates was generated by including women who had at least one cervical specimen within two calendar date windows separated by ≥ 6 months. The captured records were then further screened for predetermined eligibility criteria (described below). This method yielded a sufficient sample size of women eligible for inclusion as controls in the study but failed to yield sufficient cases for the study. As the hospital implemented the comprehensive electronic medical record system, queries were conducted of this system to capture all women with a diagnosis of high-grade cervical dysplasia using the medical billing diagnostic codes ICD-9-CM 622.1 (Dysplasia of cervix uteri) and ICD-9-CM 233.1 (Carcinoma in situ of cervix/CIN3) for dates prior to October 2015, and ICD-10-CM N87 (Dysplasia of cervix uteri) and ICD-10-CM D06 (carcinoma in situ of cervix uteri/CIN3) for dates including and after October 2015. The electronic medical record of each woman returned in the query was then reviewed to identify women with prior LGCIN diagnosis. Complete history of gynecological health encounters was reviewed for each study candidate, including results of cervical cytology, HPV co-testing, cervical histopathology, p16 immunostaining and any treatment interventions (cervical conization [cone], loop electrosurgical excision procedure [LEEP], or other surgical procedure) performed.

The study baseline visit was defined as the clinical encounter at which a woman was determined to have biopsy-proven LGCIN/CIN1 on histopathology. Women for whom a LGCIN diagnostic biopsy was not available in pathology archives were excluded. Women were also considered ineligible for the study if they were known to be HIV seropositive, if they had a history of high-grade cervical dysplasia or cervical cancer that pre-dated the baseline visit, if they had other gynecological comorbidities or if they lacked a cervix due to prior hysterectomy. A candidate who passed this initial screening was included as a case in the study if she had biopsy-confirmed high-grade cervical dysplasia (grade 3 cervical intraepithelial neoplasia, CIN3, HGCIN) in the vicinity (within two clock positions) of the baseline LGCIN biopsy. Further stringency was added by eliminating candidates with concurrent low-grade and high-grade CIN diagnoses at the study baseline visit. Women were included in the control cohort if they had cytological evidence of cervical health following the baseline visit in the absence of documentation of treatment (cone, LEEP, etc.). Further stringency was added by excluding candidates with excessive tissue removal by biopsy and those with insufficient clinical encounters to determine a clear clinical outcome. Women with persistent LGCIN were excluded from both cohorts.

### MicroRNA expression analysis

The formalin-fixed, paraffin-embedded tissue biopsy corresponding to the baseline visit LGCIN diagnosis for each case and control subject was obtained from pathology archives. Total RNA including small RNA component was isolated from three 5-micron slices of tissue using the miRNeasy FFPE kit (Qiagen, Germantown, Maryland, USA) according to the manufacturer’s protocol. Total RNA extracts were shipped on dry ice to LC Sciences, LLC (Houston, Texas, USA) for microRNA microarray profiling. Quality control tests were run on each specimen upon arrival and specimens that failed QC were excluded from further analysis. Comprehensive microRNA microarray profiling services included probes for all human microRNAs (2632 mature miRNAs) annotated in miRbase 22 (Griffiths-Jones et al. 2006) with additional small non-coding RNA controls. Raw data were background-subtracted and normalized using cyclic locally weighted scatterplot smoothing (LOWESS) pair-wise regression (Bolstad et al. 2003). In preparation for clustering and differential expression analyses, additional data adjustment included filtering, log2 transformation, gene centering and normalization. Differential expression analysis compared miRNA expression values in cases to those of controls using t-test. Hierarchical clustering analysis was performed using average linkage and Euclidean distance metric, and results were visualized using TIGR Multiple Experiment Viewer software (the Institute for Genomic Research). Dot plot diagrams were created and analyzed in GraphPad Prism (v.7). Average normalized miRNA expression values were calculated, and Student’s t-test was used to compare cases to controls. Alpha level *p* ≤ 0.05 was considered statistically significant.

### Predicted miRNA target search

Potential mRNA targets of the miRNAs of interest were predicted using TargetScan 7.2 (Agarwal et al. 2015). Due to the high potential for false positives, the results were limited to a cumulative weight context score of < − 1.0. Predicted targets were further validated using additional online target prediction algorithms miRDB, miRWalk, and miRMap (Chen and Wang 2020; Sticht et al. 2018; Vejnar and Zdobnov 2012). MirPathDB2.0 was utilized to identify gene ontology pathways enriched for miRNA:targets discovered in the dataset (Kehl et al. 2020). Dysregulated miRNAs were input into the database and unions were analyzed for all pathway databases MiRPathDB2.0 contained, which included Kyoto Encyclopedia of Genes and Genomes (KEGG), Gene Ontology (GO), Wikipathways, and Reactome. Heat maps were generated with pathways enriched for ≥ 10 miRNAs (KEGG) or ≥ 16 miRNAs (GO), and all miRNAs with ≥ 1 predicted target, included in the map.

## Results

### Case–control cohort

**Fig. 1:**
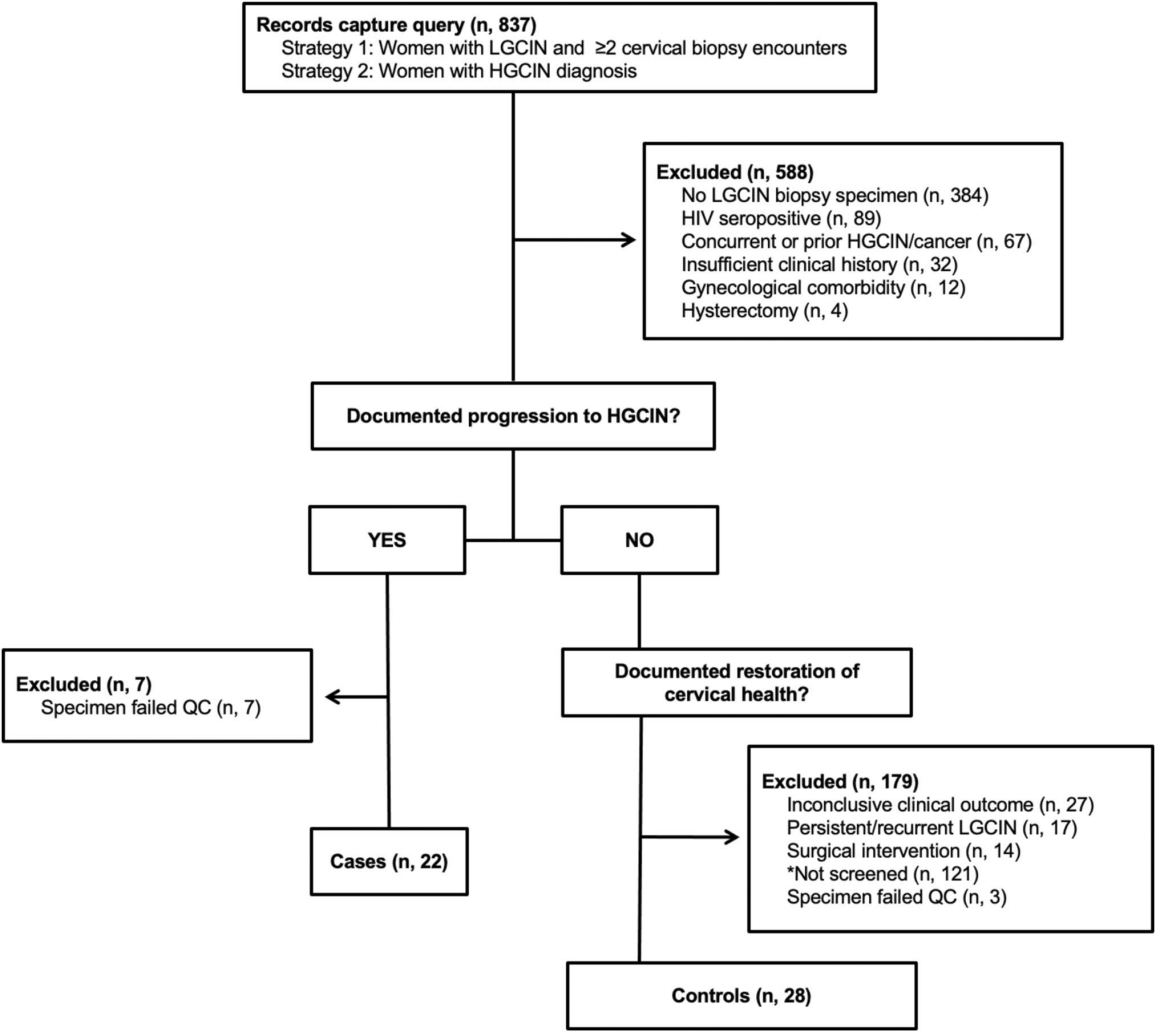
Diagram of LGCIN cohort enrollment

Women participating in cervical cancer screening at the University Medical Center hospital in New Orleans, LA with a diagnosis of LGCIN (*n* = 837) were screened for inclusion in the study. The study enrollment diagram is shown in Fig. 1. Women with low-grade CIN and a subsequent diagnosis of high-grade CIN were further screened for inclusion as cases in the study, for a total enrollment of 29 women. Women with low-grade CIN and subsequent clinical indications of restoration to healthy cervical tissue without evidence of medical intervention were included as controls in the study, for a total enrollment of 31 women. The final dataset included only those samples with high-quality miRNA microarray results (22 cases, 28 controls). Characteristics of the cohort are reported in Table 1. All participants with available HPV results had tested HPV positive. Most participants (74%) entered colposcopy follow-up with a prior atypia or low-grade squamous intraepithelial lesion (LGSIL) cytology result. Cases were marginally older than controls (mean age 34 vs 29, *p* = 0.049). Results of p16 immunostaining supported the HGCIN diagnosis in all cases for which immunostaining study was performed. The average observation period for controls (39 months) exceeded the average time to progression for cases (21 months).

**Table 1:** Characteristics of cohort

Characteristic	Controls	Cases	*p* value
Sample size	28	22	–
Age (avg, range)	29 (21–49)	34 (23–62)	**0.049**
Baseline cytology result (*n*, %)			0.512
ASCUS	8 (28.6)	4 (18.2)	
ASC-H	2 (7.1) (53.7)	2 (9.1)	
LGSIL	15 (0)	10 (45.5)	
LGSIL + H	0 (0)	1 (4.6)	
HGSIL	0 (0)	1 (4.6)	
HR HPV test (*n*, %)			1.00
Result available	14 (50)	11 (50)	
Positive	14 (100)	11 (100)	
P16 staining result (*n*, %)			–
Result available	–	12 (54.5)	
Positive		12 (100)	
Time to resolution (months)			–
2nd normal test result (avg, SEM)	28 (5.3)	–	
Total observation period (avg, SEM)	39 (7.4)		
Time to progression (months, avg, SEM)	–	21 (4.4)	–

Bold font indicates statistical signifance *p* ≤ 0.05

Missing results: Baseline cytology, 4 (2 controls, 2 cases); HPV test, (14 controls, 11 cases); HGCIN p16 staining, 10. Reasons for missing results include the test was not done or the test was performed at a different clinical site and results were not recorded in the EMR. Unpaired *t*-test was used to compare difference in mean age. Cytology and HPV test were compared as categorical variables by Chi square and Fisher’s Exact test (significance, *p* < 0.05)

### Differential expression of miRNAs in low-grade CIN stratified by clinical outcome

**Fig. 2:**
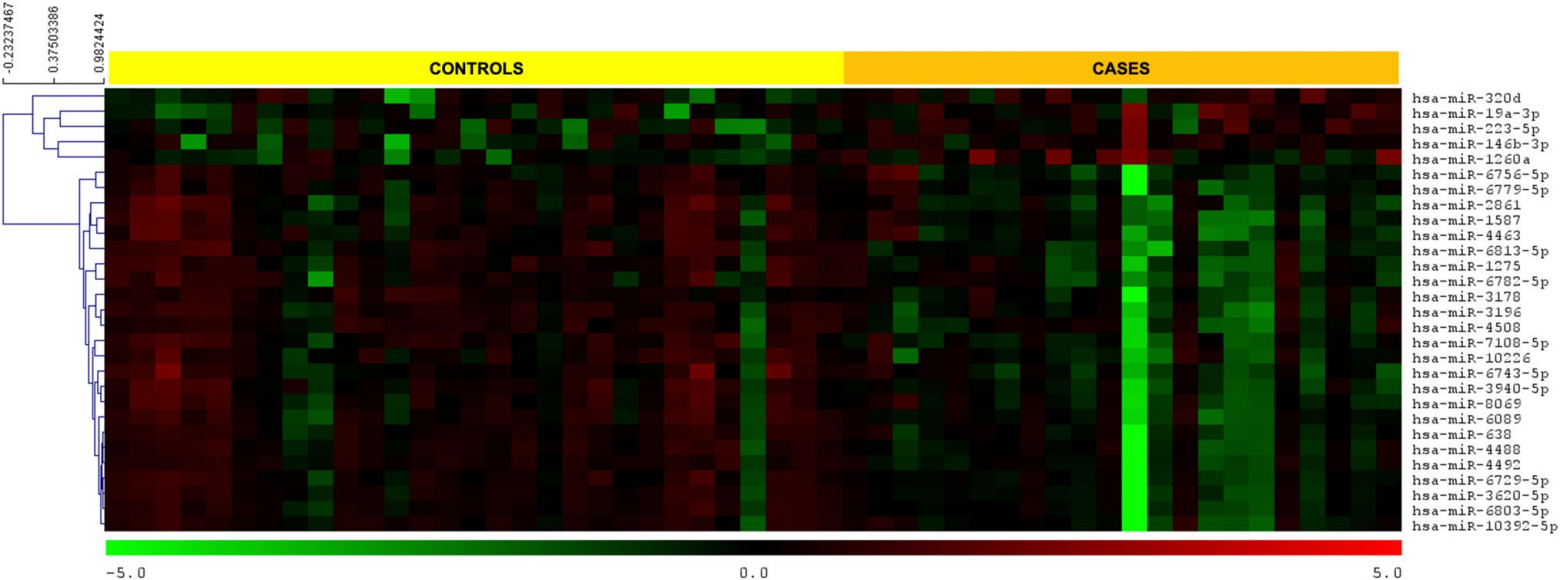
Heat map of microRNAs significantly dysregulated (*p* < 0.01) in LGCIN that progressed to HGCIN (cases) compared to LGCIN that resolved (controls). Green gradient represents downregulation and red gradient represents upregulation of the miRNA

**Fig. 3:**
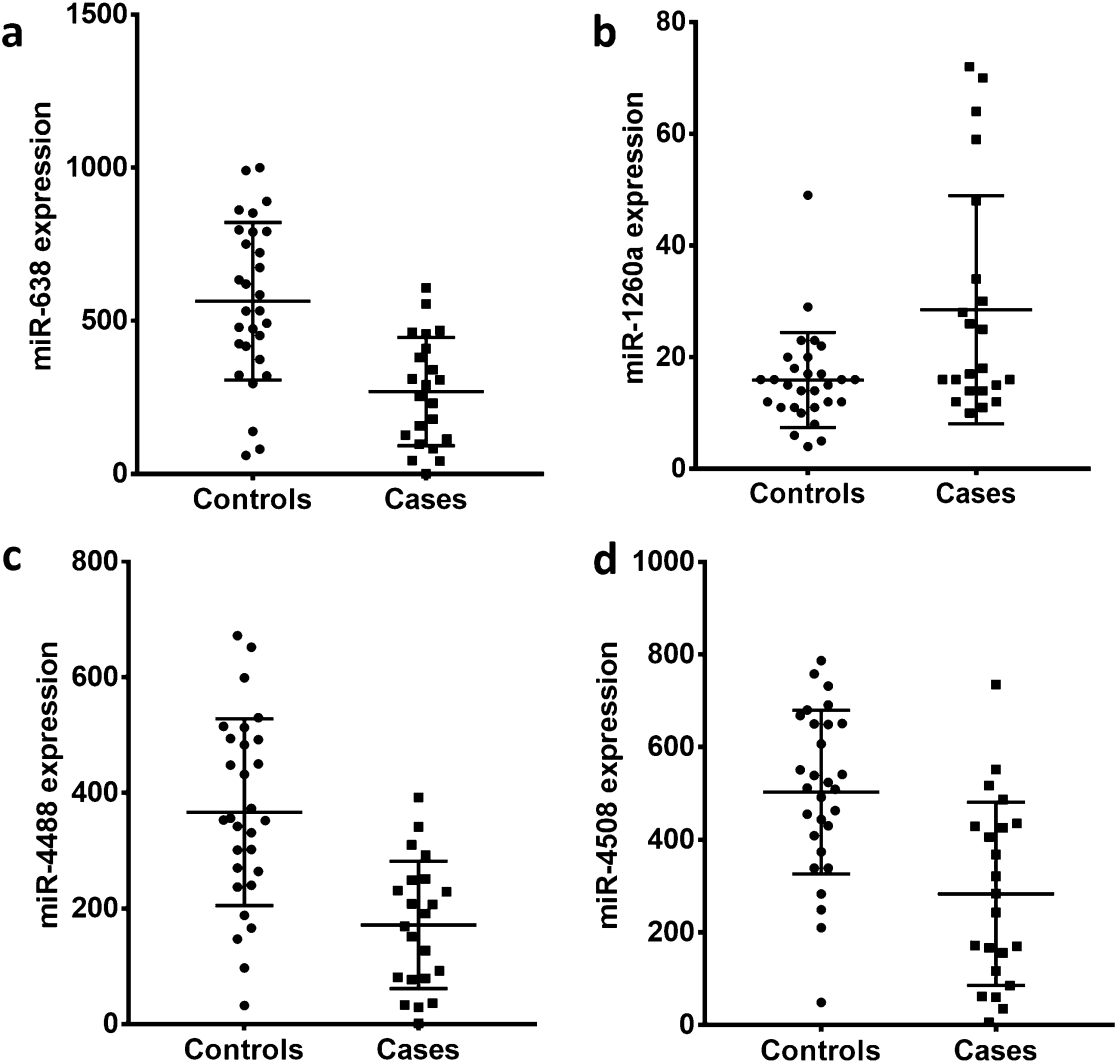
Dysregulation of microRNAs in LGCIN that progressed to HGCIN. Normalized average fluorescence intensity of replicate miRNA probes are plotted for each case (LGCIN that progressed) and control (LGCIN that resolved) subject. Middle bars represent the group mean and whiskers represent the standard deviation. Two-tailed Student’s t-test was used to compare the means of the groups and *p* < 0.05 was considered significant. Panel **A** miR-638; panel **B** miR-1260a; panel **C** miR-4488; panel **D** miR-4508

We performed comprehensive miRNA microarray analysis for expression of 2632 known human miRNAs in low-grade cervical biopsy tissues. Differential expression analysis of miRNA profiles obtained from cases and controls revealed 29 miRNAs to be significantly dysregulated (*p* < 0.01; Fig. 2). Of these, 24 were downregulated while only 5 miRNAs were found to be upregulated in cases compared to controls (See Supplemental Information 1). Next, we plotted the normalized expression values for each case and control patient for four of the dysregulated miRNAs (Fig. 3). On average, increased expression of miR-1260a and decreased expression of miR-638, miR-4508, and miR-4488 was observed in biopsies of LGCIN that progressed compared to biopsies of LGCIN that resolved.

### Gene ontology analysis to identify pathways enriched in progressive LGCIN

**Fig. 4:**
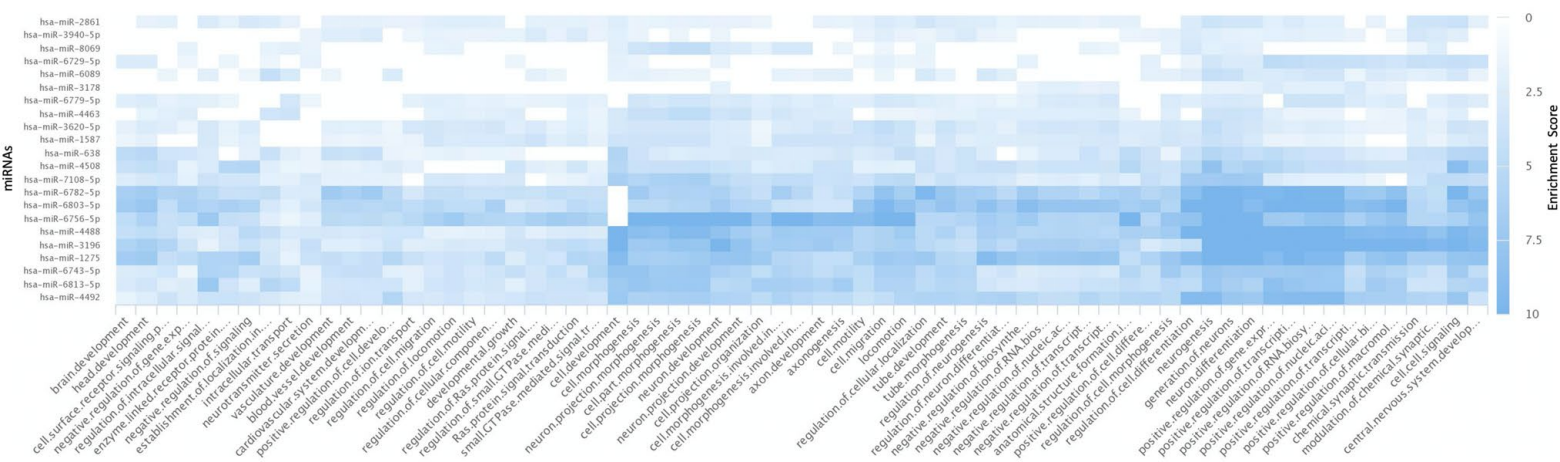
Heat map of the gene ontology biological processes pathways enriched for predicted mRNA targets of the 24 miRNAs significantly downregulated (*p* < 0.01) in LGCIN that progressed to HGCIN. Blue gradient represents enrichment score. Pathways were included if they met a threshold of ≥ 16 predicted miRNA:mRNA target pairs within the pathway

To identify cell signaling pathways potentially activated in cervical dysplasia progression, we performed pathway enrichment analysis using miRPathDB 2.0 (Kehl et al. 2020). MicroRNAs (*n* = 24) found to be downregulated in progressive LGCIN were input into the discovery algorithm, and predicted unions were analyzed using both the KEGG and Gene Ontology pathway databases. Gene Ontology discovery identified 67 pathways enriched with putative mRNA targets of the downregulated miRNAs (Fig. 4 and Supplemental Information 2). Pathways related to tumor growth and progression were identified among the enriched pathways, including the GO terms transcription activation, signal transduction (small GTPase and Ras), angiogenesis, and metastasis (cellular motility, migration and locomotion). KEGG pathway analysis confirmed enrichment of miRNA targets in Ras signaling and revealed other well-known cancer related pathways including Wnt, ErBb and MAPK signaling (see Supplemental Information 3). These data suggest that miRNA dysregulation during LGCIN contributes to the progression of LGCIN to HGCIN by promoting and enhancing pathways traditionally associated with cancer.

### Predicted transcript targets of dysregulated microRNAs

In the KEGG pathway analysis, Wnt signaling emerged as the most targeted pathway with 14 miRNAs predicted to regulate the pathway (Additional file 3). Specific transcript targets within the Wnt signaling pathway were explored by comparing target predictions of four databases: TargetScan, miRDB, miRmap, and miRWalk (Agarwal et al. 2015; Chen and Wang 2020; Sticht et al. 2018; Vejnar and Zdobnov 2012). Several members of the Wnt family of proteins were predicted by multiple databases to be targeted by one or more miRNA(s) in our dataset. WNT6 was predicted to be targeted by miR-2861. MicroRNAs miR-1275 and miR-3620-5p were strongly predicted to target WNT5A, and WNT7B was a predicted target of miR-1275 and miR6756-5p. Interestingly, miR-4492 was highly predicted to target TCF7L1, a transcription factor activated by Wnt signaling. Reduced expression of miRNAs collectively targeting Wnt signaling family members suggests the possibility that Wnt signaling is activated in the progression of low-grade cervical dysplasia.

## Discussion

In this report, we have identified differential expression of miRNAs that may be exploited to triage women with LGCIN for appropriate clinical management. These miRNAs may also have a mechanistic role in dysplasia progression. MicroRNA-638 was found to be downregulated in women with LGCIN that progressed to HGCIN. This miRNA is a widely studied tumor suppressor found to be downregulated in cervical cancer as well as leukemia, glioma, melanoma, and cancers of the lung (non-small cell), colon, liver, stomach, breast, esophagus, oral cavity and endometrium (Chong et al. 2021; Wei et al. 2017; Yuan et al. 2020). MicroRNA-638 has been shown to target apoptotic pathways, cell proliferative pathways and metastatic pathways (Chong et al. 2021). Conversely, miR-1260a was upregulated in women with LGCIN that progressed. Upregulation of miR-1260a has been linked to many cancers including cancers of the sex organs (ovarian cancer in women and prostate cancer in men) (Ghafour et al. 2021; Said et al. 2018), and based on studies of the closely related miRNA miR-1260b, may promote invasion/metastasis (Morita et al. 2020; Xu et al. 2018). The dysregulation of miR-638 and miR-1260a at the early stages of cervical dysplasia may facilitate advancement to high-grade cervical dysplasia by causing dysregulation of pathways involved in oncogenesis.

Other differentially expressed miRNAs identified in our study have potential to be involved in oncogenesis; however, their dysregulation is inconsistent across cancer biology studies. MicroRNA-4508, found to be downregulated in progressive LGCIN in our study, was reported to be downregulated in bladder cancer (Sabo et al. 2020). Likewise, Nagy et al. analyzed colorectal precancerous lesions and found miR-4508 to be downregulated twofold as the severity of the polyps increased from tubular to tubulovillous adenoma (Nagy et al. 2017). However, miR-4508 was shown to be upregulated in breast cancer (Boo et al. 2016). Like miR-4508, miR-4488 also demonstrates conflicting dysregulation patterns in cancer. Consistent with our finding of miR-4488 downregulation in progressive LGCIN, miR-4488 has been found to be downregulated in liver and breast cancer (Cui et al. 2014; Zheng et al. 2020; Matamala et al. 2015). However, upregulation of miR-4488 was reported in esophageal adenocarcinoma and colon cancer (Ames et al. 2017; Drahos et al. 2016). Furthermore, Yuan et al. reported 2.85-fold log2 induction of miR-4488 in cervical cancer tissue compared to healthy tissue from the same patient (Yuan et al. 2020). It is possible that there may be a bimodal pattern of miR-4488 dysregulation during the progression of cervical dysplasia to cancer that permits different oncogenic processes at different stages of cancer development.

It is important to note that no single miRNA emerged as a clear, stand-alone predictor of LGCIN progression in our study. This is not surprising given that multiple miRNAs target single mRNA transcripts; multiple transcripts are involved in individual signaling pathways, and individual miRNAs regulate multiple mRNA transcripts. This complex and dynamic relationship between miRNAs and their targets must be taken into consideration when investigating the role of miRNAs in the pathophysiology of disease. Our gene ontology analysis that included the entire network of downregulated miRNAs and their targets revealed Wnt signaling as a putative dysregulated pathway in LGCIN that progressed to HGCIN, which is consistent with reports of aberrant Wnt signaling in cervical carcinoma (Perez-Plasencia et al. 2007; Liang et al. 2016; Zhao et al. 2019). Previous reports have implicated overexpression of Wnt5A and Wnt-11 in cervical cancer invasion and metastasis (Lin et al. 2014; Wei et al. 2016); our analysis suggested possible acquisition of cell motility and migratory traits in LGCIN that progressed, potentially through deregulation of Wnt signaling mediators secondary to loss of miRNA-mediated translational suppression. The enhancement of these putative signaling pathways and the phenotypic consequences of miRNA dysregulation in progressive LGCIN are areas ripe for further empirical investigation.

Our study has notable limitations. First, we relied upon the subjective evaluation of the histopathology grade of baseline LGCIN specimens that was documented in the medical record by the clinical pathologist. Notably, misclassification of baseline histopathology may account for some individuals that progressed, underscored by the one subject in the case group that had HSIL cytology result at baseline (Table 1). We argue that whether or not the LGCIN was an accurate diagnosis, it was in fact the documented diagnosis on the clinical chart that informed the course of clinical management for women in our cohort. Thus, if our study included cases that were misdiagnosed at baseline in addition to those that truly progressed from LGCIN at baseline to HGCIN at the end of the study period, then this reflects the real-world clinical experience, and our molecular findings are pragmatic from the perspective of clinical application. Indeed, the potential for misclassification of subjectively scored histopathology grade highlights the need for more objective molecular tests to better identify women who will ultimately receive a diagnosis of HGCIN.

The retrospective study design restricted our ability to account for known modulators of cervical dysplasia risk, including HPV genotype and lifestyle factors such as smoking, sexual activity, and hormonal contraceptive use (Nagelhout et al. 2021; Cancer 2009; Cancer et al. 2007; Iversen et al. 2021). However, we excluded women with gynecologic comorbidities and human immunodeficiency virus infection, which were predicted to influence miRNA expression patterns. This study also suffered from small sample size, in part due to inconsistent intervals between clinical encounters that obscured definitive clinical outcomes for many women with LGCIN diagnoses. Large prospective cohort studies that can systematically account for confounding factors and execute consistent follow-up intervals are needed to improve the predictive value of miRNAs for LGCIN outcomes. In addition, the limited amount of residual tissue remaining in the archived specimens precluded further confirmatory studies such as repeat histopathology and/or p16 immunostaining to verify the documented clinical diagnosis, in situ hybridization for dysregulated miRNAs or immunostaining for markers of putatively dysregulated signaling pathways. Despite these limitations, the data presented offers novel insights into early neoplastic changes that may lead to cervical dysplasia progression.

## Conclusions

MiRNA dysregulation observed in our study was consistent with prior reports in cancer tissues, including cervical cancer. Our data suggest that early changes in miRNA expression may aid in LGCIN progression by altering cancer-associated pathways within the dysplastic tissue. Further studies are necessary to understand the functional role of dysregulated miRNAs and their cognate targets in cervical dysplasia advancement. Increased understanding of the biological processes associated with LGCIN progression will create opportunities for innovative pharmaceutical interventions and objective, prognostic-focused adjunct tests that will improve clinical management of this common women’s health issue.

## Supplementary Information

Below is the link to the electronic supplementary material.

Supplementary file1 (PDF 833 KB)

Supplementary file2 (XLSX 16 KB)

Supplementary file3 (PDF 602 KB)

## Data Availability

The miRNA expression data generated for this study are publicly available in Gene Expression Omnibus (GEO) at GSE171597 (Edgar et al. 2002).

## References

[CR1] Agarwal V, Bell GW, Nam JW, Bartel DP (2015). Predicting effective microRNA target sites in mammalian mRNAs. Elife.

[CR2] Ames HM, Yuan M, Vizcaino MA, Yu W, Rodriguez FJ (2017). MicroRNA profiling of low-grade glial and glioneuronal tumors shows an independent role for cluster 14q32.31 member miR-487b. Mod Pathol.

[CR3] Baseman JG, Koutsky LA (2005). The epidemiology of human papillomavirus infections. J Clin Virol.

[CR4] Bolstad BM, Irizarry RA, Astrand M, Speed TP (2003). A comparison of normalization methods for high density oligonucleotide array data based on variance and bias. Bioinformatics.

[CR5] Boo L, Ho WY, Ali NM, Yeap SK, Ky H, Chan KG, Yin WF, Satharasinghe DA, Liew WC, Tan SW, Ong HK, Cheong SK (2016). MiRNA transcriptome profiling of spheroid-enriched cells with cancer stem cell properties in human breast MCF-7 cell line. Int J Biol Sci.

[CR6] Cancer, International Collaboration of Epidemiological Studies of Cervical (2009). Cervical carcinoma and sexual behavior: collaborative reanalysis of individual data on 15,461 women with cervical carcinoma and 29,164 women without cervical carcinoma from 21 epidemiological studies. Cancer Epidemiol Biomarkers Prev.

[CR7] Appleby P, Beral V, Berrington de Gonzalez A, Colin D, Franceschi S, Goodhill A, Green J, Peto J, Plummer M, Sweetland S, Cancer, International Collaboration of Epidemiological Studies of Cervical (2007). Cervical cancer and hormonal contraceptives: collaborative reanalysis of individual data for 16,573 women with cervical cancer and 35,509 women without cervical cancer from 24 epidemiological studies. Lancet.

[CR8] Chen Y, Wang X (2020). miRDB: an online database for prediction of functional microRNA targets. Nucleic Acids Res.

[CR9] Chen PS, Su JL, Hung MC (2012). Dysregulation of microRNAs in cancer. J Biomed Sci.

[CR10] Chesson HW, Ekwueme DU, Saraiya M, Watson M, Lowy DR, Markowitz LE (2012). Estimates of the annual direct medical costs of the prevention and treatment of disease associated with human papillomavirus in the United States. Vaccine.

[CR11] Cheung TH, Man KN, Yu MY, Yim SF, Siu NS, Lo KW, Doran G, Wong RR, Wang VW, Smith DI, Worley MJ, Berkowitz RS, Chung TK, Wong YF (2012). Dysregulated microRNAs in the pathogenesis and progression of cervical neoplasm. Cell Cycle.

[CR12] Chong ZX, Yeap SK, Ho WY (2021). Dysregulation of miR-638 in the progression of cancers. Pathol Res Pract.

[CR13] Craig MP, Rajakaruna S, Paliy O, Sajjad M, Madhavan S, Reddy N, Zhang J, Bottomley M, Agrawal S, Kadakia MP (2020). Differential MicroRNA Signatures in the Pathogenesis of Barrett's Esophagus. Clin Transl Gastroenterol.

[CR14] Cui ZH, Shen SQ, Chen ZB, Hu C (2014). Growth inhibition of hepatocellular carcinoma tumor endothelial cells by miR-204-3p and underlying mechanism. World J Gastroenterol.

[CR15] Drahos J, Schwameis K, Orzolek LD, Hao H, Birner P, Taylor PR, Pfeiffer RM, Schoppmann SF, Cook MB (2016). MicroRNA profiles of barrett's esophagus and esophageal adenocarcinoma: differences in glandular non-native epithelium. Cancer Epidemiol Biomarkers Prev.

[CR16] Edgar R, Domrachev M, Lash AE (2002). Gene Expression Omnibus: NCBI gene expression and hybridization array data repository. Nucleic Acids Res.

[CR17] Curry SJ, Krist AH, Owens DK, Barry MJ, Caughey AB, Davidson KW, Doubeni CA, Epling JW, Kemper AR, Kubik M, Landefeld CS, Mangione CM, Phipps MG, Silverstein M, Simon MA, Tseng CW, Wong JB, Force, U. S. Preventive Services Task (2018). Screening for cervical cancer: US preventive services task force recommendation statement. JAMA.

[CR18] Ghafour AA, Odemis DA, Tuncer SB, Kurt B, Saral MA, Erciyas SK, Erdogan OS, Celik B, Saip P, Yazici H (2021). High expression level of miR-1260 family in the peripheral blood of patients with ovarian carcinoma. J Ovarian Res.

[CR19] Gray NM, Sharp L, Cotton SC, Masson LF, Little J, Walker LG, Avis M, Philips Z, Russell I, Whynes D, Cruickshank M, Woolley CM, Tombola group (2006). Psychological effects of a low-grade abnormal cervical smear test result: anxiety and associated factors. Br J Cancer.

[CR20] Griffiths-Jones S, Grocock RJ, van Dongen S, Bateman A, Enright AJ (2006). miRBase: microRNA sequences, targets and gene nomenclature. Nucleic Acids Res.

[CR21] Henk HJ, Insinga RP, Singhal PK, Darkow T (2010). Incidence and costs of cervical intraepithelial neoplasia in a US commercially insured population. J Low Genit Tract Dis.

[CR22] Insinga RP, Glass AG, Rush BB (2004). The health care costs of cervical human papillomavirus–related disease. Am J Obstet Gynecol.

[CR23] Iversen L, Fielding S, Lidegaard O, Hannaford PC (2021). Contemporary hormonal contraception and cervical cancer in women of reproductive age. Int J Cancer.

[CR24] Kehl T, Kern F, Backes C, Fehlmann T, Stockel D, Meese E, Lenhof HP, Keller A (2020). miRPathDB 2.0: a novel release of the miRNA pathway dictionary database. Nucleic Acids Res.

[CR25] Liang J, Zhou H, Peng Y, Xie X, Li R, Liu Y, Xie Q, Lin Z (2016). β-Catenin expression negatively correlates with WIF1 and predicts poor clinical outcomes in patients with cervical cancer. Biomed Res Int.

[CR26] Lin L, Liu Y, Zhao W, Sun B, Chen Q (2014). Wnt5A expression is associated with the tumor metastasis and clinical survival in cervical cancer. Int J Clin Exp Pathol.

[CR27] Matamala N, Vargas MT, Gonzalez-Campora R, Minambres R, Arias JI, Menendez P, Andres-Leon E, Gomez-Lopez G, Yanowsky K, Calvete-Candenas J, Inglada-Perez L, Martinez-Delgado B, Benitez J (2015). Tumor microRNA expression profiling identifies circulating microRNAs for early breast cancer detection. Clin Chem.

[CR28] McCaffery KJ, Irwig L, Turner R, Chan SF, Macaskill P, Lewicka M, Clarke J, Weisberg E, Barratt A (2010). Psychosocial outcomes of three triage methods for the management of borderline abnormal cervical smears: an open randomised trial. BMJ.

[CR29] Morita T, Fujiwara T, Yoshida A, Uotani K, Kiyono M, Yokoo S, Hasei J, Kunisada T, Ozaki T (2020). Clinical relevance and functional significance of cell-free microRNA-1260b expression profiles in infiltrative myxofibrosarcoma. Sci Rep.

[CR30] Musselwhite LW, Oliveira CM, Kwaramba T, de Paula Pantano N, Smith JS, Fregnani JH, Reis RM, Mauad E, Vazquez FL, Longatto-Filho A (2016). Racial/ethnic disparities in cervical cancer screening and outcomes. Acta Cytol.

[CR31] Nagelhout G, Ebisch RM, Van Der Hel O, Meerkerk GJ, Magnee T, De Bruijn T, Van Straaten B (2021). 'Is smoking an independent risk factor for developing cervical intra-epithelial neoplasia and cervical cancer? A systematic review and meta-analysis. Expert Rev Anticancer Ther.

[CR32] Nagy ZB, Wichmann B, Kalmar A, Galamb O, Bartak BK, Spisak S, Tulassay Z, Molnar B (2017). Colorectal adenoma and carcinoma specific miRNA profiles in biopsy and their expression in plasma specimens. Clin Epigenetics.

[CR33] Oberg AL, French AJ, Sarver AL, Subramanian S, Morlan BW, Riska SM, Borralho PM, Cunningham JM, Boardman LA, Wang L, Smyrk TC, Asmann Y, Steer CJ, Thibodeau SN (2011). miRNA expression in colon polyps provides evidence for a multihit model of colon cancer. PLoS One.

[CR34] Perez-Plasencia C, Vazquez-Ortiz G, Lopez-Romero R, Pina-Sanchez P, Moreno J, Salcedo M (2007). Genome wide expression analysis in HPV16 cervical cancer: identification of altered metabolic pathways. Infect Agent Cancer.

[CR35] Sabo AA, Birolo G, Naccarati A, Dragomir MP, Aneli S, Allione A, Oderda M, Allasia M, Gontero P, Sacerdote C, Vineis P, Matullo G, Pardini B (2020). Small non-coding RNA profiling in plasma extracellular vesicles of bladder cancer patients by next-generation sequencing: expression levels of miR-126–3p and piR-5936 increase with higher histologic grades. Cancers (basel).

[CR36] Said R, Garcia-Mayea Y, Trabelsi N, Setti Boubaker N, Mir C, Blel A, Ati N, Paciucci R, Hernandez-Losa J, Rammeh S, Derouiche A, Chebil M, Leonart L, Ouerhani S (2018). Expression patterns and bioinformatic analysis of miR-1260a and miR-1274a in Prostate Cancer Tunisian patients. Mol Biol Rep.

[CR37] Sharp L, Cotton S, Cochran C, Gray N, Little J, Neal K, Cruickshank M, For the Tombola Group (2009). After-effects reported by women following colposcopy, cervical biopsies and LLETZ: results from the TOMBOLA trial. BJOG.

[CR38] Spence AR, Goggin P, Franco EL (2007). Process of care failures in invasive cervical cancer: systematic review and meta-analysis. Prev Med.

[CR39] Stachowiak M, Flisikowska T, Bauersachs S, Perleberg C, Pausch H, Switonski M, Kind A, Saur D, Schnieke A, Flisikowski K (2017). Altered microRNA profiles during early colon adenoma progression in a porcine model of familial adenomatous polyposis. Oncotarget.

[CR40] Sticht C, De La Torre C, Parveen A, Gretz N (2018). miRWalk: an online resource for prediction of microRNA binding sites. PLoS One.

[CR41] Vejnar CE, Zdobnov EM (2012). MiRmap: comprehensive prediction of microRNA target repression strength. Nucleic Acids Res.

[CR42] Wei H, Wang N, Zhang Y, Wang S, Pang X, Zhang S (2016). Wnt-11 overexpression promoting the invasion of cervical cancer cells. Tumour Biol.

[CR43] Wei H, Zhang JJ, Tang QL (2017). MiR-638 inhibits cervical cancer metastasis through Wnt/*β*-catenin signaling pathway and correlates with prognosis of cervical cancer patients. Eur Rev Med Pharmacol Sci.

[CR44] Wilting SM, Snijders PJ, Verlaat W, Jaspers A, van de Wiel MA, van Wieringen WN, Meijer GA, Kenter GG, Yi Y, le Sage C, Agami R, Meijer CJ, Steenbergen RD (2013). Altered microRNA expression associated with chromosomal changes contributes to cervical carcinogenesis. Oncogene.

[CR45] Xu L, Xu X, Huang H, Ma Z, Zhang S, Niu P, Chen Y, Ping J, Lu P, Yu C, Min L, Chen J, Dai L, Dong S (2018). MiR-1260b promotes the migration and invasion in non-small cell lung cancer via targeting PTPRK. Pathol Res Pract.

[CR46] Yuan Y, Shi X, Li B, Peng M, Zhu T, Lv G, Liu L, Jin H, Li L, Qin D (2020). Integrated analysis of key microRNAs/TFs/mRNAs/ in HPV-positive cervical cancer based on microRNA sequencing and bioinformatics analysis. Pathol Res Pract.

[CR47] Zhao L, Wang L, Zhang C, Liu Z, Piao Y, Yan J, Xiang R, Yao Y, Shi Y (2019). E6-induced selective translation of WNT4 and JIP2 promotes the progression of cervical cancer via a noncanonical WNT signaling pathway. Signal Transduct Target Ther.

[CR48] Zheng X, Lu S, He Z, Huang H, Yao Z, Miao Y, Cai C, Zou F (2020). MCU-dependent negative sorting of miR-4488 to extracellular vesicles enhances angiogenesis and promotes breast cancer metastatic colonization. Oncogene.

