# Supplementary material for: MicroRNA expression associated with low-grade cervical intraepithelial neoplasia outcomes
Source: J Cancer Res Clin Oncol. 2023 Jul 8;149(13):11969–78. doi: 10.1007/s00432-023-05023-3 (PMC10465678; doi:10.1007/s00432-023-05023-3)
Supplement: Supplementary file 1 — Supplementary file1 (PDF 833 KB) [file 432_2023_5023_MOESM1_ESM.pdf]

Supplemental Information 1. microRNAs significantly dysregulated in LGCIN that progressed to HGCIN. Fold-Δ, fold change in normalized average fluorescent signal intensity. Negative values represent downregulation of miRNA in cases compared to controls. Differential expression of these miRNAs was statistically significant ($p<0.01$).

**microRNA**	**Fold-Δ**	**p-value**
hsa-miR-3196	-1.80	1.18×10^{-3}
hsa-miR-4508	-1.78	2.41×10^{-3}
hsa-miR-4488	-2.10	3.34×10^{-3}
hsa-miR-6089	-2.18	5.47×10^{-3}
hsa-miR-638	-2.10	6.01×10^{-3}
hsa-miR-6803-5p	-2.50	9.49×10^{-3}
hsa-miR-6813-5p	-2.27	1.24×10^{-4}
hsa-miR-3940-5p	-2.73	1.40×10^{-4}
hsa-miR-2861	-2.15	4.42×10^{-4}
hsa-miR-4463	-1.95	7.23×10^{-4}
hsa-miR-1587	-2.03	1.05×10^{-3}
hsa-miR-1275	-1.91	1.11×10^{-3}
hsa-miR-7108-5p	-2.13	1.36×10^{-3}
hsa-miR-6743-5p	-2.68	1.40×10^{-3}
hsa-miR-1260a	1.87	2.16×10^{-3}
hsa-miR-10226	-1.83	2.90×10^{-3}
hsa-miR-320d	1.57	3.91×10^{-3}
hsa-miR-3620-5p	-2.49	3.92×10^{-3}
hsa-miR-223-5p	4.00	4.18×10^{-3}
hsa-miR-8069	-2.19	4.21×10^{-3}
hsa-miR-3178	-1.84	4.34×10^{-3}
hsa-miR-6782-5p	-2.44	4.82×10^{-3}
hsa-miR-146b-3p	5.00	4.89×10^{-3}
hsa-miR-4492	-2.12	4.90×10^{-3}
hsa-miR-6729-5p	-2.73	5.68×10^{-3}
hsa-miR-19a-3p	2.67	7.44×10^{-3}
hsa-miR-10392-5p	-1.84	7.89×10^{-3}
hsa-miR-6756-5p	-1.53	8.22×10^{-3}
hsa-miR-6779-5p	-1.88	9.30×10^{-3}

MicroRNA expression associated with low-grade cervical intraepithelial neoplasia outcomes
Journal of Cancer Research and Clinical Oncology

A. Winters[1], A. Berry[1], T. Dewenter[2], N. Chowdhury[1], K. Wright[1] and J. Cameron*[1,3]

Departments of [1]Microbiology, Immunology & Parasitology and [2]Pathology and [3]The Stanley S. Scott Cancer Center, Louisiana State University Health Sciences Center, New Orleans, LA, USA

*Corresponding Author: Jennifer E. Cameron, Ph.D., jcame2@lsuhsc.edu
