# Supplementary material for: MicroRNA expression associated with low-grade cervical intraepithelial neoplasia outcomes
Source: J Cancer Res Clin Oncol. 2023 Jul 8;149(13):11969–78. doi: 10.1007/s00432-023-05023-3 (PMC10465678; doi:10.1007/s00432-023-05023-3)
Supplement: Supplementary file 3 — Supplementary file3 (PDF 602 KB) [file 432_2023_5023_MOESM3_ESM.pdf]

Supplemental Information 3. Heat map of the KEGG pathways enriched for predicted mRNA targets of the 24 miRNAs significantly downregulated (P<0.01) in LGCIN that progressed to HGCIN.

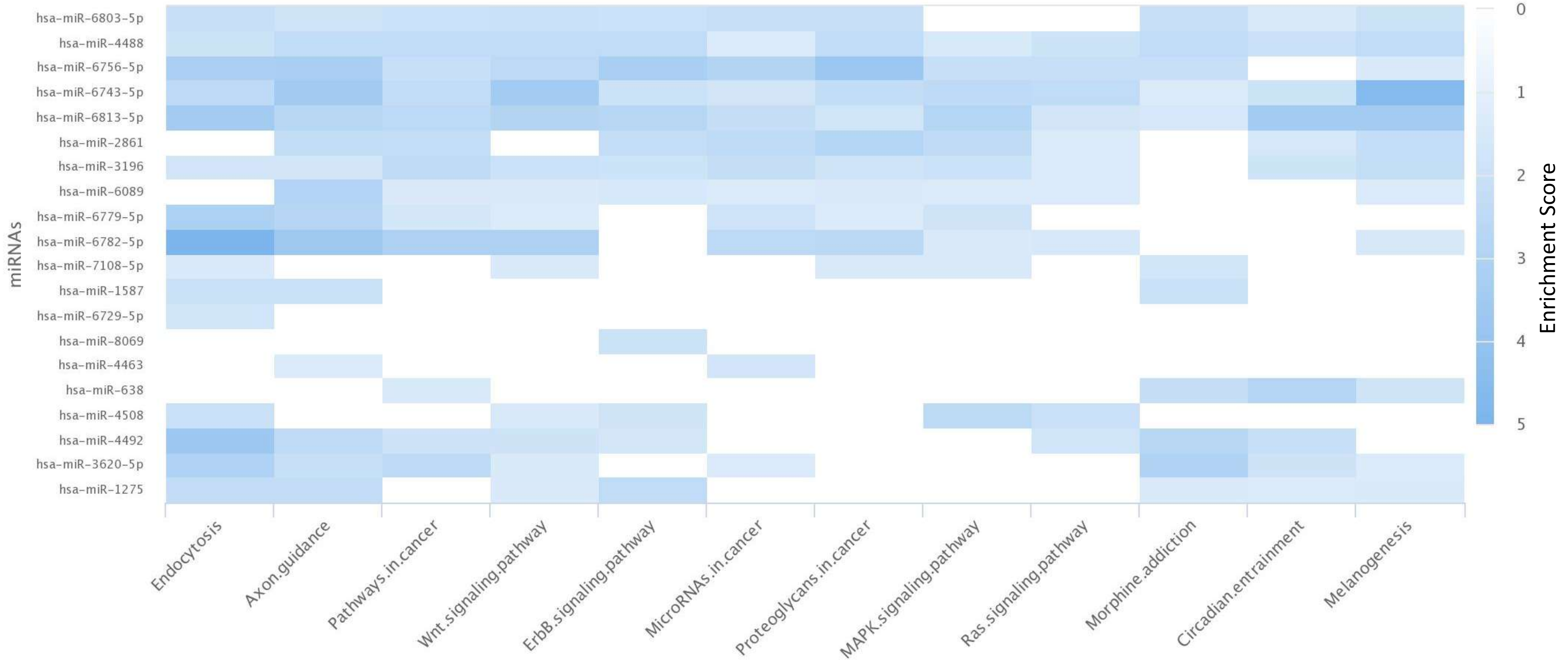

MicroRNA expression associated with low-grade cervical intraepithelial neoplasia outcomes *Journal of Cancer Research and Clinical Oncology*
A. Winters[1], A. Berry[1], T. Dewenter[2], N. Chowdhury[1], K. Wright[1] and J. Cameron*[1,3] *Corresponding author: Jennifer E. Cameron, Ph.D., jcame2@lsuhsc.edu
Departments of [1]Microbiology, Immunology & Parasitology and [2]Pathology and [3]The Stanley S. Scott Cancer Center, Louisiana State University Health Sciences Center, New Orleans, LA, USA
